# The Psychological Impact of COVID-19 on Hospital Staff

**DOI:** 10.5811/westjem.2020.11.49015

**Published:** 2021-02-08

**Authors:** Sameer Hassamal, Fanglong Dong, Sunita Hassamal, Carol Lee, Dotun Ogunyemi, Michael M. Neeki

**Affiliations:** * Arrowhead Regional Medical Center, Department of Psychiatry, Colton, California; † Western University of Health Sciences, Graduate College of Biomedical Sciences, Pomona, California; ‡ Arrowhead Regional Medical Center, Department of Internal Medicine, Colton, California; § Arrowhead Regional Medical Center, Department of Emergency Medicine, Colton, California; ¶ Arrowhead Regional Medical Center, Graduate Medical Education, Colton, California

## Abstract

**Introduction:**

The coronavirus 2019 (COVID-19) pandemic has created a mental health crisis among hospital staff who have been mentally and physically exhausted by uncertainty and unexpected stressors. However, the mental health challenges and complexities faced by hospital staff in the United States has not been fully elucidated. To address this gap, we conducted this study to examine the prevalence and correlates of depression and anxiety among hospital staff in light of the COVID-19 pandemic.

**Methods:**

The design is a single-center, cross-sectional, online survey evaluating depression and anxiety among all hospital employees (n = 3,500) at a safety-net hospital with a moderate cumulative COVID-19 hospitalization rate between April 30–May 22, 2020. We assessed depression with the Patient Health Questionnaire-9. Anxiety was measured with the Generalized Anxiety Disorder-7 scale. Logistic regression analyses were calculated to identify associations with depression and anxiety.

**Results:**

Of 3,500 hospital employees, 1,246 (36%) responded to the survey. We included 1,232 individuals in the final analysis. Overall, psychological distress was common among the respondents: 21% and 33% of staff reported significant depression and anxiety, respectively, while 46% experienced overwhelming stress due to COVID-19. Notably, staff members overwhelmed by the stress of COVID-19 were seven and nine times more likely to suffer from depression and anxiety, respectively. In addition to stress, individuals with six to nine years of work experience were two times more likely to report moderate or severe depression compared to those with 10 or more years of work experience. Moreover, ancillary staff with direct patient contact (odds ratio [OR] 8.9, confidence interval (CI), 1.46, 173.03) as well as administrative and ancillary staff with indirect patient contact (OR 5.9, CI, 1.06, 111.01) were more likely to be depressed than physicians and advanced providers.

**Conclusion:**

We found that a considerable proportion of staff were suffering from psychological distress. COVID-19-associated depression and anxiety was widespread among hospital staff even in settings with comparatively lower COVID-19 hospitalization rates. Ancillary staff, administrative staff, staff with less job experience, and staff overwhelmed by the stress of COVID-19 are particularly susceptible to negative mental health outcomes. These findings will help inform hospital policymakers on best practices to develop interventions to reduce the mental health burden associated with COVID-19 in vulnerable hospital staff.

## INTRODUCTION

In December 2019, a cluster of idiopathic pneumonia cases linked to a seafood market emerged in Wuhan, China.^1^ Genomic sequencing analysis revealed that a novel coronavirus strain, severe acute respiratory syndrome coronavirus 2 (SARS-CoV-2), was the causative agent that resulted in coronavirus disease 2019 (COVID-19).^2^ Epidemiological investigations determined that SARS-CoV-2 is highly contagious and primarily spread through person-to-person contact.^3^ The virus spread at an alarming rate infecting millions of people, and as a result governments around the world enforced lockdown measures to mitigate community transmission. On March 11, 2020, the World Health Organization declared COVID-19 a pandemic, signaling that the viral illness was a global emergency.^4^

The COVID-19 pandemic has created a parallel mental health crisis in the United States (US). Preliminary results indicate that prevalence rates of depression and anxiety have tripled in the US since the inception of the pandemic.^5^ In particular, healthcare workers have been psychologically burdened by high levels of work-related COVID-19 stress.^6^,^7^ Emerging data suggests that up to 50% of healthcare workers will experience moderate to severe depression and anxiety.^8^ Moreover, healthcare workers are at a heightened risk of developing stress-related disorders due to experiencing or witnessing human suffering and trauma. Initial studies project that up to 60% of healthcare workers treating patients with COVID-19 will develop symptoms of acute stress disorder.^9^ Factors contributing to mental health distress range from psychological and social stressors intrinsic to a novel pandemic to shortages of personal protective equipment.^10^ The demands of COVID-19 will undoubtedly further strain the mental health wellbeing of healthcare workers. For this reason, expert panels have requested a call for action to understand the psychological effects of COVID-19.^11^

A handful of observational studies have examined the psychological consequences of COVID-19. A meta-analysis reported that the prevalence of anxiety ranged between 22.6–36.3% and depression between 16.5–48.3% in healthcare workers.^12^ The studies included in the meta-analysis primarily focused on healthcare workers providing care in regions of China severely affected by the pandemic. It is important to bear in mind that mental health outcomes among healthcare workers may differ based on region, infection rate, and COVID-19-associated hospitalization rates. Nevertheless, it is likely that the untoward psychological effects of COVID-19 are systemic across the entire health workforce. To date, little is known about the mental health needs of healthcare workers in light of the unprecedented pressures faced by hospitals. To address this gap, we sought in this study to understand the scope of depression and anxiety among staff at a safety-net hospital with a moderate cumulative COVID-19 hospitalization rate. We aimed to determine the prevalence of depression and anxiety, and to elucidate associations between sociodemographic variables, depression, and anxiety.

**Population Health Research Capsule**

What do we already know about this issue?

The COVID-19 pandemic has created a parallel mental health crisis among hospital staff who are experiencing burnout and stress-related disorders.

What was the research question?

Our goal was to examine the prevalence and correlates of depression and anxiety among staff at a general medical hospital.

What was the major finding of the study?

Overall, 21% and 33% of staff reported significant depression and anxiety, respectively, especially ancillary and administrative staff and those with less job experience,.

How does this improve population health?

Psychological interventions are needed at the hospital organizational level to improve mental health outcomes and the wellbeing of staff.

## METHODS

### Study Design and Participants

The study, which was approved by the institutional review board at our institution, is a cross-sectional, anonymous, Internet-based survey conducted at a safety-net hospital in San Bernardino County, California, between April 30–May 22, 2020 During the study period, the number of confirmed COVID-19 cases doubled from 2,058 to 4,146 in the county, and a total of 146 patients were treated for COVID-19 at the hospital site. We developed the survey using SurveyMonkey (San Mateo, CA), and the survey web link was emailed to all hospital employees (n = 3500) biweekly. All staff employed by the hospital were asked to participate. Inclusion criteria were as follows: 1) older than 18 years; 2) hospital staff; 3) willing and able to give informed consent; and 4) able to complete the survey in English.

### Measures

Voluntary electronic informed consent was provided by participants prior to beginning the survey. The survey was anonymous, and no identifying information such as name, email address, or Internet-provider information was collected. Participants were permitted to withdraw from the survey at any time. Occupation was classified into four groups: 1) physicians and advanced providers; 2) nursing staff; 3) ancillary staff with direct patient contact; and 4) administrative and ancillary staff with indirect patient contact. Age was classified into three groups: 1) millennials (20–39 years); 2) generation X (40–55 years); and 3) baby boomers (56–75 years). Zhu and colleagues found that 10 or more years of work experience was a risk factor for COVID-related depression and anxiety among healthcare workers. Accordingly, in our study we categorized years of work experience as follows: 1) zero to five years, 2) six to nine years, and 3) ≥10 years.^13^

To measure perceived stress participants were asked, “Have you been overwhelmed by the stress of the COVID-19 pandemic?” (Y/N). Studies have validated that perceptions of stress can be measured by asking individuals how overwhelmed they are by a situation.^14^ To assess whether staff were front-line or second-line we asked, “Have you been in contact with a patient either suspected to have COVID-19 or confirmed to have COVID-19?” (Y/N). Staff who answered “Yes” were classified as front-line and those who answered “No” were classified as second-line. The Patient Health Questionnaire (PHQ)-9 and General Anxiety Disorder (GAD)-7 were completed to measure depressive and anxious symptomatology, respectively.

### Outcomes

The primary outcome measures were the PHQ-9 score (range, 0–27), and GAD-7 score (range, 0–21). Patient Health Questionnaire-9: This self-reported measure consists of nine questions to measure the frequency of depressive symptoms over the prior two weeks on a four-point Likert-scale ranging from 0 (not at all) to 3 (nearly every day). The scores are interpreted as follows: normal (0–4); mild (5–9); moderate (10–14); moderately severe (15–19); and severe (20–27). A PHQ-9 score ≥10 is 88% sensitive and 88% specific for a diagnosis of major depression.^15^ Accordingly, we grouped PHQ-9 scores into two categories: PHQ-9 score <10; PHQ-9 score≥10.

General Anxiety Disorder-7: A self-reported measure that consists of seven questions to measure the severity of anxiety symptoms over the prior two weeks on a four-point Likert-scale ranging from 0 (not at all) to 3 (nearly every day). The scores are interpreted as follows: normal (0–4); mild (5–9); moderate (10–14); and severe (15–21). The GAD-7 is a well-validated tool for assessing anxiety disorders; generalized anxiety disorder (sensitivity of 89%, specificity of 82%); panic disorder (sensitivity of 74%, specificity of 81%); social anxiety disorder (sensitivity of 72%, specificity of 80%); and post-traumatic stress disorder (sensitivity of 66%, specificity of 81%).^16^ Accordingly, GAD-7 scores were grouped into two categories: GAD-7 score <8; GAD-7 score ≥ 8.

#### Statistical Analysis

We conducted all statistical analyses using the SAS software for Windows version 9.3 (SAS Institute, Cary, NC). Descriptive statistics are presented as means and standard deviations for continuous variables, and frequencies and proportions for categorical variables. Chi-square statistics were conducted comparing whether staff were overwhelmed by the stress of COVID-19 between sociodemographic factors and scores on the PHQ-9 and GAD-7. Logistic regression analyses were conducted to examine predictors for a PHQ-9 score ≥10 and a GAD-7 score ≥8. These predictors included occupation, age, gender, years in current position, being overwhelmed by the stress of COVID-19, and being in contact with a patient either suspected to have COVID-19 or confirmed to have COVID-19. All statistical analyses were two-sided. *p*-value ≤ 0.05 was considered to be statistically significant.

## RESULTS

Out of 3,500 staff 1246 (36%) responded to the survey. Among the 1,246 staff who completed the survey, eight refused to participate and six did not indicate whether they consented to participate in the survey. We included a total of 1232 staff in the final analysis. Descriptive statistics are summarized in Table 1. Overall, 21% of respondents were depressed, 33% had anxiety, and 46% were overwhelmed by the stress of COVID-19. Chi-square analysis was conducted to compare staff stressed and not stressed by COVID-19. The results of the chi-square analysis are presented in Table 2. Occupation (*P*<0.001), gender (*P*<0.001), front-line vs second-line staff (*P* = 0.013), age (*P* = 0.036), depression severity (*P* <0.001), and anxiety severity (*P*<0.001) impacted stress perceptions.

We calculated the first logistic regression analysis to examine predictors for staff with a PHQ-9 score ≥10. The results of the logistic regression analysis are presented in Table 3. Ancillary staff with direct patient contact (odds ratio [OR] 8.9; confidence interval [CI], 1.46,173.03), and administrative and ancillary staff with indirect patient contact (OR 5.9; CI,1.06, 111.01) were more likely to be depressed than physicians and advanced providers. Compared to staff with 10 or more years of work experience, staff working six to nine years were more likely to be depressed (OR 2.08; CI, 1.24, 3.5). Stress was also associated with depression; staff overwhelmed by the stress of COVID-19 were more likely to report significant depressive symptoms compared to staff not overwhelmed by the stress of COVID-19 (OR, 7.06; CI, 4.8, 10.63).

We calculated the second logistic regression analysis to examine predictors for staff with a GAD-7 score ≥8. The results of the logistic regression analysis are presented in Table 4. Staff overwhelmed by the stress of COVID-19 were more likely to experience significant anxiety compared to staff not overwhelmed by the stress of COVID-19 (OR 9; CI, 6.49, 12.65).

## DISCUSSION

We examined the prevalence and correlates of depression and anxiety among hospital staff during the COVID-19 pandemic. This is one of the largest studies in the US examining psychological consequences among hospital staff during the COVID-19 pandemic. Overall, 21% and 33% of staff reported significant depression and anxiety, respectively. These findings support that depression and anxiety are pervasive among hospital staff even in settings with comparatively lower COVID-19 hospitalization rates. Published studies from the epicenter of the pandemic have reported slightly higher rates of depression and anxiety.^8^ A similar study examining the psychological effects of COVID-19 reported comparable rates of anxiety among healthcare workers caring for patients in New York City at the peak of COVID-19.^9^

Our hospital is not considered a COVID-19 designated center, and the results are conceivably more reflective of the general mental health experience of hospital staff. However, it is important to highlight that we may not be able to draw broad inferences considering the low survey-response rate and single-center design. By virtue of the low survey-response rate, it may be expected that our data overstates the prevalence of depression and anxiety because of non-response bias. Furthermore, a limitation of the single-center design is difficulty extrapolating the results to other settings and populations.

Our results indicate that certain hospital staff members were prone to more severe depressive symptoms. Specifically, ancillary and administrative staff were especially burdened with greater depressive symptomatology. On the contrary, physicians and advanced providers experienced less depression compared to ancillary staff providing direct patient care. Similarly to our findings, Zhu and colleagues found that physicians were less likely to report distress compared to medical technicians.^13^ A potential explanation is that ancillary staff directly interacting with patients are mentally exhausted by greater workloads and closer contact time with patients, evoking a fear of contagion.^17^ Hospital staff providing indirect-care functions are also increasingly burdened by challenges as never before the pandemic. In our study, administrative and ancillary staff with indirect patient contact reported more severe depressive symptoms than physicians and advanced providers. The etiology of depression is multifactorial, and it is plausible that distress among staff not directly interacting with patients is situational and triggered by institutional concerns, lack of social support, and isolation.^18^ Altogether, hospital staff are navigating high-intensity stressful situations, which may potentially induce adverse psychological changes.^19^

Stress is highly prevalent among individuals with depression and anxiety.^20^ In our study, 46% of staff experienced overwhelming stress during the COVID-19 pandemic. As anticipated, staff stressed over COVID-19 experienced considerable anxiety and depression. Moreover, staff with substantial anxiety and depression reported heightened stress about the COVID-19 outbreak. Causal relationships could not be fully elucidated as this was a cross-sectional study. However, these findings support that almost half of the hospital staff respondents experienced a substantial psychological burden during the COVID-19 crisis. Importantly, research has shown that pandemic-related stress has deleterious effects on health-related quality of life.^21^ In view of these findings, there is a critical need for hospital systems to develop interventions to mitigate adverse mental health consequences and to improve the psychological resiliency of staff.

Another significant finding was that staff with 10 or more years of work experience reported lower levels of depression than staff with six to nine years of work experience. Factors that may explain why staff with more years of work experience reported less depressive symptoms include the following:1) practical experience navigating complex situations; 2) experience managing patients during prior epidemics; 3) the development of adaptive coping skills over time; 4) robust social supports; and 5) job security. These results are in contrast to a similar study by Zhu and colleagues who found that increasing years of work experience was associated with more severe depressive symptoms among healthcare workers during the COVID-19 pandemic.^13^ Discrepancies between work experience and depression may be attributed to confounding variables that were not accounted for; our broad inclusion criteria consisting of a wide range of hospital occupations; and cultural differences.

## LIMITATIONS

There are several limitations of this study: 1) the data is cross-sectional and we could not establish causality; 2) selection bias as we used a web-based survey that was voluntary; 3) self-selection bias as more females voluntarily participated than males; 4) there was no data on participants’ mental health prior to the COVID-19 outbreak; 5) the low survey-response rate; 6) the lack of screening questionnaires specific for acute stress disorder; 7) the results of the screening questionnaires were not confirmed with comprehensive diagnostic assessments; 8) the results are from a single center and might not be generalizable; and 9) our findings may not be representative of the entire hospital work force as a greater proportion of staff with indirect compared to direct patient contact participated in the study.

## CONCLUSION

In this study, we found that depression and anxiety were pervasive among hospital staff. Our results identified specific groups of hospital staff experiencing depression and anxiety. Ancillary staff, administrative staff, and staff with less job experience are particularly vulnerable to negative mental health outcomes. The COVID-19 pandemic has been overwhelming, and a considerable proportion of staff reported stress as a result of COVID-19. Moreover, elevated stress levels were associated with clinically significant depression and anxiety. If left untreated, psychological distress can have long-term negative consequences that adversely lead to burnout and poor patient care. Therefore, it is imperative that hospital systems develop and implement screening resources to evaluate for stress, depression, and anxiety among staff. Early detection and assistance may potentially reduce the distress associated with COVID-19 and promote psychological well-being.

## Figures and Tables

**Table 1:** Hospital staff characteristics.

Variable	Frequency	Percent
Gender		
Female	959	77.8%
Male	273	22.2%
Age		
Millennials (20–39 years)	500	44.2%
Generation X (40–55 years)	426	37.6%
Baby boomers (56–75 years)	206	18.2%
Occupation		
Admin + ancillary staff with indirect patient care	491	42.2%
Ancillary staff with direct medical care	68	5.8%
Nursing staff	463	39.8%
Physician + advanced practitioner	142	12.2%
Years In current position?		
0–5 years	506	49.0%
6–9 years	157	15.2%
10+ years	370	35.8%
Overwhelmed by the stress of COVID-19?		
No	624	54.2%
Yes	527	45.8%
Contact with a patient suspected or confirmed to have COVID-19?		
No	632	55.2%
Yes	514	44.9%
PHQ-9		
PHQ-9 score <10	872	79.3%
PHQ-9 score ≥10	227	20.7%
GAD-7		
GAD7 score<8	743	67.6%
GAD7 score≥8	357	32.5%

*PHQ-9*, Patient Health Questionnaire; *GAD-7*, General Anxiety Disorder.

**Table 2:** Chi-square analysis comparing staff overwhelmed and not overwhelmed by the stress of COVID-19.

Factors	Overwhelmed by the stress of COVID-19		*p*-value

	No	Yes	
Gender			<0.001
Female	449 (50.4%)	442 (49.6%)	
Male	175 (67.3%)	85 (32.7%)	
Age			0.036
Millennials (20–39 years)	253 (51.4%)	239 (48.6%)	
Generation X (40–55 years)	225 (53.6%)	195 (46.4%)	
Baby boomers (56–75 years)	126 (62.1%)	77 (37.9%)	
Occupation			<0.001
Admin + ancillary staff with indirect patient contact	254 (52.3%)	232 (47.7%)	
Ancillary staff with direct patient contact	35 (53%)	31 (47%)	
Nursing	234 (51.2%)	223 (48.8%)	
Physician + advanced practitioner	100 (70.9%)	41 (29.1%)	
Years in current position			0.723
0–5	259 (51.5%)	244 (48.5%)	
6–9	78 (50%)	78 (50%)	
10+	197 (53.5%)	171 (46.5%)	
Contact with a patient suspected or confirmed to have COVID-19?			0.013
Yes	258 (50.2%)	256 (49.8%)	
No	363 (57.5%)	268 (42.5%)	
PHQ-9 score			<0.001
PHQ-9 score<10	547 (62.7%)	325 (37.3%)	
PHQ-9 score 10+	46 (20.3%)	181 (79.7%)	
GAD-7 score			<0.001
GAD-7 score<8	518 (69.7%)	225 (30.3%)	
GAD-7 score 8+	76 (21.3%)	281 (78.7%)	

*PHQ-9*, Patient Health Questionnaire; *GAD-7*, General Anxiety Disorder.

**Table 3:** Logistic regression analysis to examine predictors for a patient health questionnaire-9 ≥10.

Predictors	Adjusted odds ratio	*p*-value
Occupation		
Admin + ancillary staff with indirect patient contact	5.9 (1.06,111.01)	0.042
Ancillary staff with direct patient contact	8.9 (1.46,173.03)	0.015
Nursing	3.85 (0.69,72.44)	0.139
Physician + advanced practitioner	Reference	
Age		
Millennial (20–39 years)	1.1 (0.65,1.91)	0.723
Generation X (40–55 years)	1.03 (0.63,1.71)	0.898
Baby boomers (56–75 years)	Reference	
Gender		
Female vs male	1.07 (0.68,1.7)	0.784
Years in current position		
0–5 years	1.18 (0.76,1.83)	0.463
6–9 years	2.08 (1.24,3.5)	0.006
10+ years	Reference	
Overwhelmed by the stress of COVID-19? (Y/N)	7.06 (4.8,10.63)	<0.0001
Contact with a patient suspected or confirmed to have COVID-19? (Y/N)	1.33 (0.92,1.93)	0.132

**Table 4:** Logistic regression analysis to examine predictors for a generalized anxiety disorder-7 score ≥8.

Predictors	Adjusted Odds Ratio	*p*-value
Occupation		
Admin + Ancillary staff with indirect patient contact	2.61 (0.72,12.51)	0.153
Ancillary staff with direct patient contact	3.82 (0.94,19.74)	0.062
Nursing	1.95 (0.54,9.33)	0.324
Physician + Advanced Practitioner	Reference	
Age		
Millennial (20–39 years)	1.17 (0.72,1.92)	0.520
Generation X (40–55 years)	1.04 (0.67,1.63)	0.848
Baby boomers (56–75 years)	Reference	
Gender		
Female vs male	1.24 (0.82,1.88)	0.319
Years in current position		
0–5 years	0.91 (0.62,1.35)	0.645
6–9 years	0.88 (0.54,1.44)	0.620
10+ years	Reference	
Overwhelmed by the stress of COVID-19? (Y/N)	9 (6.49,12.65)	<0.0001
Contact with a patient suspected or confirmed to have COVID-19? (Y/N)	1.29 (0.92,1.82)	0.141
